# Comprehensive Genomic Profiling of Circulating Cell-Free DNA Distinguishes Focal *MET* Amplification from Aneuploidy in Diverse Advanced Cancers

**DOI:** 10.3390/curroncol28050317

**Published:** 2021-09-26

**Authors:** Yuichi Kumaki, Steve Olsen, Mitsukuni Suenaga, Tsuyoshi Nakagawa, Hiroyuki Uetake, Sadakatsu Ikeda

**Affiliations:** 1 Department of Specialized Surgery, Tokyo Medical and Dental University, Tokyo 113-8519, Japan; suenaga.srg2@tmd.ac.jp (M.S.); nakagawa.srg2@tmd.ac.jp (T.N.); 2 Guardant Health Japan Corp, Tokyo 105-0022, Japan; solsen@guardantamea.com; 3 Department of Clinical Research, National Hospital Organization Disaster Medical Center, Tokyo 190-0014, Japan; 7110srg2@tmd.ac.jp; 4 Center for Innovative Cancer Treatment, Tokyo Medical and Dental University, Tokyo 113-8519, Japan; ikeda.canc@tmd.ac.jp; 5 Moores Cancer Center, University of California, San Diego, CA 92037, USA

**Keywords:** *MET*, ctDNA, cfDNA, liquid biopsies, focal amplification, aneuploidy

## Abstract

Amplification (amp) of *MET* can be observed in cases of focal gene copy number gain, such as *MET*-driven amp, or with a gain of chromosome 7, such as aneuploidy. Several studies have shown that only high-level focal *MET* amp (*MET*/CEP7 ratio ≥5) is oncogenic, with such tumors responding to targeted therapy. However, there are few reports on how to distinguish between focal amplification and aneuploidy using next-generation sequencing (NGS). A total of 1025 patients with advanced solid tumors (typically pre-treated) were tested with a non-invasive comprehensive cfDNA NGS panel (Guardant360) from July 2014 to June 2019. Since bioinformatics upgrades of Guardant360 were undergoing in September 2018, focal *MET* amp was determined by our independent algorithm using the cohorts tested before September 2018 (291 patients), and validation was performed in the remaining cohort (734 patients). *MET* alterations (alts) associated with aberrant signaling were found in 110 patients (10.7%) among nine different cancer types, most commonly in non-small cell (12.2%, 62/510) and small cell (33.3%, 3/9) lung cancers, gastroesophageal cancer (19.4%, 7/36), and prostate adenocarcinoma (15.6%; 5/32). Among 291 patients tested before September 2018, 37 (12.7%) had *MET* alts. Among these, 24 (64.9%) had amps, 5 (13.5%) had exon 14 skipping, and 13 (35.1%) had single nucleotide variants (SNVs). Co-alterations, such as amp + SNVs, were found in four samples (10.8%). Among 24 *MET* amps, 29.2% (7/24) were focal according to our algorithm. *MET* copy number was significantly higher with focal amp compared to non-focal amp (mean copy number 3.26 vs. 2.44, respectively, *p* = 0.00304). In 734 patients tested after September 2018, our definition of focal *MET* amp was detected in 4.2% (31/734). Overall, focal amplification based on our algorithm was 3.7% (=38/1025). This study describes an approach to distinguish focal and non-focal *MET* amplification using comprehensive genomic profiling of cfDNA in advanced cancer patients. Focal *MET* amp accounted for ~30% of all *MET* amp, which was found in 3.7% of patients with diverse cancers and was associated with a higher plasma copy number. Clinical studies are warranted to assess the clinical utility of targeted therapies for tumors with focal *MET* amplification detected by NGS of cfDNA.

## 1. Introduction

MET, also known as c-MET, is a receptor tyrosine kinase that regulates many physiological processes, including proliferation, scattering, morphogenesis, and survival. The binding of its ligand, hepatocyte growth factor (HGF), to the MET receptor leads to the activation of several downstream signaling pathways, including RAS-ERK/MAPK, PI3K-AKT, or PLCgamma-PKC [1]. *MET* is a proto-oncogene that encodes MET, and activating *MET* mutations have been reported in diverse carcinomas [2]. 

*MET* alterations (alts), such as *MET* amplification (amp), exon 14 skipping, and single nucleotide variants (SNVs), can be oncogenic drivers. Furthermore, activating *MET* alts may also be acquired as a mechanism of resistance to tyrosine kinase inhibitors. *MET* exon 14 skipping has been described as a driver mutation in non-small cell lung cancer (NSCLC) [3], and approximately 3% of NSCLC patients are reported to have *MET* exon 14 skipping [4]. Recently, *MET* amp has also been described as oncogenic, and studies using fluorescence in situ hybridization (FISH) have found *MET* amp in up to ~5% of patients with NSCLC or gastric adenocarcinoma [5]. However, a phase I study reported that patients harboring *MET* amp did not respond to therapy with a c-MET inhibitor [2]. 

*MET* amp can occur from focal gene copy number gain of the *MET* gene alone (focal amplification), co-amplification with adjacent genes in chromosome 7q such as *CDK6* and *BRAF* (non-focal amplification), or gain of chromosome 7q copy number (e.g., aneuploidy). Several studies have shown that only high-level focal *MET* amp (*MET*/CEP7 ratio ≥5), but not non-focal or lower levels of focal *MET* amp, are oncogenic and respond to targeted therapy [6,7,8]. MET inhibitor-sensitive lung cancers with high-level *MET* amp have been reported in the absence of other sensitizing *MET* alts, such as exon 14 skipping, particularly among those with higher *MET* to chromosome 7 ratios. Therefore, it is critical to distinguish between focal amplification and non-focal amplification. A typical companion diagnostic test interrogates only the target gene but may not include adjacent genes; therefore, in such cases, the distinction between *MET* focal amplification and non-focal amplification is not feasible. There are few reports on how to distinguish between focal amplification and aneuploidy using next-generation sequencing (NGS).

NGS of cell-free plasma DNA (cfDNA) from patients with advanced cancers is a validated, non-invasive technique that can be used for comprehensive genomic profiling (CGP) of tumor-derived DNA. It can detect gene mutations with a relatively good correlation with tissue sequencing. In this study, we analyzed *MET* alterations associated with aberrant *MET* signaling in 1025 patients with advanced solid tumors by using a comprehensive cfDNA NGS panel. We examined the pattern of focal and non-focal *MET* amps and established a way to investigate the prevalence of this potentially targetable alteration on NGS panels.

## 2. Materials and Methods

### 2.1. Patients

We reviewed the results of CGP from plasma of 1025 mostly pre-treated patients with advanced solid tumors. CGP of cfDNA was performed as a routine clinical practice between July 2014 and June 2019 from patients seen at institutes in Asia, Middle East, and Africa. Results from patients participating in prospective clinical trials were excluded. De-identified results were shared by Guardant Health Japan, Corp. Ethics review committee approval was granted by the institutional review board at Tokyo Medical and Dental University (G2020-021). 

### 2.2. Next-Generation Sequencing

All 1025 patients were tested with Guardant360^®^, a comprehensive cfDNA NGS panel performed at Guardant Health, Inc. (Redwood City, California), a Clinical Laboratory Improvement Amendment (CLIA)-certified and College of American Pathologists (CAP)-accredited clinical laboratory. This assay interrogates single nucleotide substitutions, indels, amplification, and gene fusions in selected genes. During the study period, several versions of the assay were employed. In each version, *MET* amplification, *MET* single nucleotide substitutions, *MET* exon 14 skipping, and amplifications of *BRAF*, *CDK6*, and *EGFR* were included. Prior to September 2018, Guardant360 reported *MET* amplification regardless of focality; after bioinformatics upgrades, only focal *MET* amplification was reported. Therefore, for purposes of the present analysis, unfiltered Guardant360 amplification results for *MET*, *BRAF*, *CDK6*, and *EGFR* were provided for all samples. Copy number variants were scored based on the baseline copy number pooled in previous CGP data at Guardant Health, Inc. The result of amplification was reported as observed copy number. 

### 2.3. Determination of Focal Amplification

**Figure 1:**
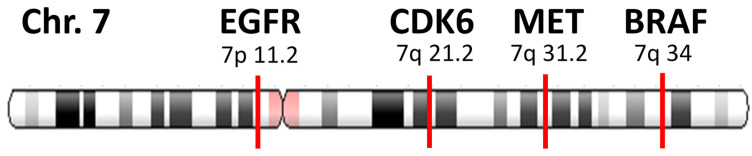
Gene location on chromosome 7. In chromosome 7, the *EGFR* gene is located in 7p 11.2, whereas the other three genes (*CDK6*, *MET*, and *BRAF*) are located in arm 7q.

**Figure 2:**
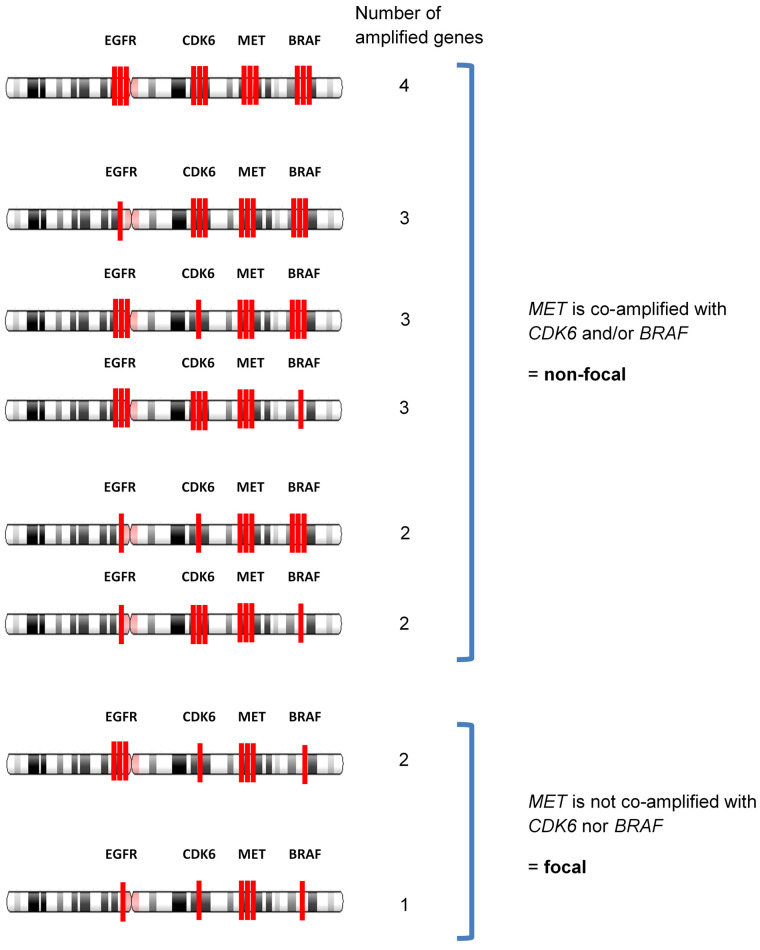
Patterns of amplified genes on chromosome 7 and definition of focal/non-focal. There are four genes on chromosome 7 for which amplification can be detected by Guardant360, and we defined *MET* focal/non-focal by the pattern of amplification of the four genes; *MET* is co-amplified with *CDK6* and/or *BRAF* = non-focal and *MET* is not co-amplified with *CDK6* nor *BRAF* = focal. *EGFR* amplification was excluded from the definition of focal/non-focal because it tended to show independent behavior.

In chromosome 7, four genes were examined in Guardant360: *EGFR* in 7p11.2, *CDK6* in 7q21.2, *MET* in 7q31.2, and *BRAF* in 7q34. (Figure 1) Focal amplification was defined as *MET* amplification without aneuploidy or an increase in the chromosome copy number itself. In this case, the *MET* gene was considered amplified if there were no co-amplification of adjacent genes, such as *CDK6* or *BRAF*. On the other hand, *MET* non-focal amplification was defined as *MET* copy number increase associated with aneuploidy, in which *MET* copy number was increased together with either *CDK6* and/or *BRAF*. For example, a sample with *MET* amplification only would be categorized as *MET* focal amplification. A sample with amplification of *MET* and *EGFR* without *CDK6* amplification would be categorized as *MET* focal amplification because *CDK6* is located between *MET* and *EGFR*, and aneuploidy could not occur without increasing the copy numbers of all three genes together. A sample with co-amplification of *MET* and *EGFR* would also be defined as focal amplification when both *CDK6* and *BRAF* genes were not co-amplified, given that *EGFR* is in a distant location (chromosome 7p). (Figure 2 and Figure S1).

In this study, additional analysis to distinguish focal and non-focal amplification was performed at an independent academic institution (Tokyo Medical and Dental University) using an original algorithm not used in the standard Guardant360 workflow. We divided the whole (1025 patients) into two cohorts; the cohorts tested before September 2018 (291 patients) was used to define focal amplification, and validation was performed in the remaining cohort (734 patients). Using the former cohort, we determined the algorithm to describe focal amplification as follows.

(a) *MET* copy number ≥2.2.
(b) *MET* gene is amplified without co-amplification of *CDK6* and *BRAF*. Co-amplification status was defined as “increased together” when the copy number of other gene (*CDK6* or *BRAF*) ≥2.2, and the difference with *MET* amplification is within +/−0.5.
(c) *MET* amplification that satisfies both (a) and (b) is defined as focal.

### 2.4. Statistical and Outcome Analysis

Patient characteristics were summarized using descriptive statistics. Medians and respective 95% confidence intervals and range were calculated whenever possible. T-test or Mann–Whitney U test was used for numerical data, and Fisher’s exact test or chi-square test was used for categorical data. We set the accepted level of significance at 0.05 (*p*-value). Statistical analysis was performed using EZR software (version 1.5.4 or later) and JMP software (version 14.2). For the validation cohort, positive percent agreement, negative percent agreement, positive predictive value, and negative predictive value were assessed using the updated Guardant360 bioinformatics pipeline results as the gold standard.

## 3. Results

### 3.1. Patient Demographic Characteristics

In 1025 total samples, the mean age at the time of testing was 62.4 years (CI 95%, 61.6–63.3). The cohort included 482 men (47.0%) and 543 women (53.0%). The most common cancer types were NSCLC (49.8%, *n* = 510), non-colorectal and non-gastroesophageal gastrointestinal cancers (“other GI cancers”) (15.4%, *n* = 158), breast cancer (9.0%, *n* = 92), and colorectal cancer (7.5%, *n* = 77). There was no statistically significant imbalance of diagnosis between the *MET* alteration and *MET* non-alteration groups (Table 1).

### 3.2. MET Alterations and Associations with Patient Characteristics

*MET* alts were defined as *MET* amplification (amp), exon 14 skipping, and non-synonymous single nucleotide variants (SNVs). In total, *MET* alts were detected by Guardant360 in 110 of 1025 patients (10.7%), which was similar to a previous report [9]. 

*MET* alts were commonly found in SCLC 33.3% (3/9), gastroesophageal cancer 19.4% (7/36), prostate cancer 15.6% (5/32), and NSCLC 12.2% (62/510) (Table 1). 

*MET* alts were found more frequently in men than in women (13.9% vs. 7.9%, *p* = 0.00234) (Table 1). This may also be due to the fact that *MET* mutations tended to be more common in prostate cancer (15.6%) than in breast (8.7%) and gynecological cancer (6.5%), although the number of cases of each was small. When analyzed by cancer type, there were significantly more *MET* alts in males than in females with non-colorectal and other GI cancers (*p* = 0.00478). However, no gender differences were observed for the frequency of *MET* alterations in other cancer types. 

### 3.3. MET Alteration Types and Focal vs. Non-Focal MET Amp

**Figure 3:**
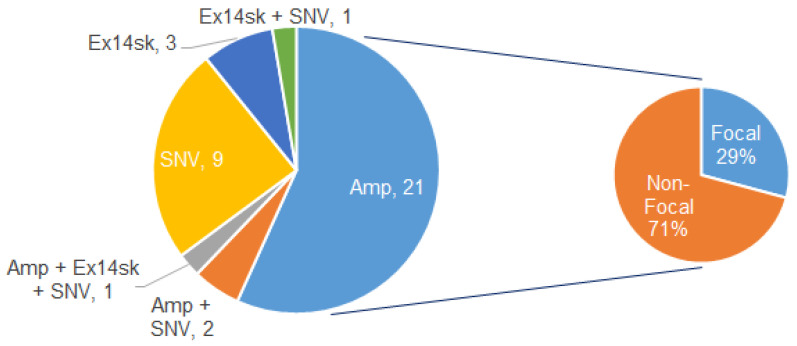
Types of *MET* alts detected in cfDNA. Among the 291 patients tested before September 2018, 37 (12.7%) had *MET* alts, according to Guardant360. In 37 patients, 24 patients (64.9%) had amps, 5 (13.5%) had exon 14 skipping, and 13 (35.1%) had SNVs. Among 24 *MET* amps, we found 17 patients (70.8%) of the non-focal *MET* amp and 7 patients (29.2%) of the focal *MET* amp, according to our algorithm.

Among 291 patients tested before Sep 2018, 37 (12.7%) had *MET* alts, according to Guardant360. Among these, 24 (64.9%) had amps, 5 (13.5%) had exon 14 skipping, and 13 (35.1%) had SNVs. Co-alterations, such as amplification + SNVs, were found in four samples (10.8%) (Figure 3).

Among the 24 samples with *MET* amp, we found several patterns of gene amplification on chromosome 7 (Table 2). Focal amplification was defined as *MET* amplification without aneuploidy or an increase in the chromosome copy number itself, and using this cohort as a test set, we established an algorithm for determining focal *MET* amplification, as described in the Methods. Using this algorithm, we found that seven cases (29.2%) were focal, and 17 (70.8%) were non-focal (Table 2, Figure 3). In the cases of focal amp, the majority (71.4%) had only *MET* amplification without amplifications of any of the other three genes from chromosome 7 included in the assay, and the minority of patients (28.6%) had co-amplification of *MET* and *EGFR*. In non-focal *MET* amplification, 82.4% of cases had co-amplification of *MET*, *CDK6*, and *BRAF*, suggesting aneuploidy. Other cases had co-amplifications of *MET* with either *CDK6* or *BRAF*.

**Figure 4:**
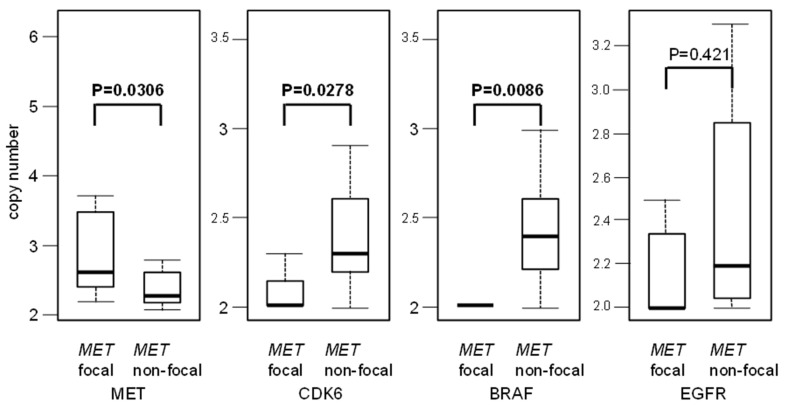
Copy number differences of four genes between *MET* focal and non-focal groups. This figure shows a comparison of the copy number sizes of the four genes in *MET* focal vs. non-focal. The copy number of the focal *MET* amp was significantly higher than that of non-focal (*p* = 0.0304). On the other hand, the copy number of *CDK6* and *BRAF* of the focal *MET* amp was significantly lower than that of the non-focal (*p* = 0.0278 and *p* = 0.0086, respectively), indicating that *CDK6* and *BRAF* are co-amplified with *MET* in non-focal. The copy number of *EGFR* was not significantly different between *MET* focal and non-focal (*p* = 0.421).

We examined the features of focal and non-focal *MET* amplifications. *MET* copy number was significantly higher with focal amp compared to non-focal amp (mean copy number 3.26 vs. 2.44, respectively, *p* = 0.00304). (Figure 4) In the case of focal *MET* amplification, *CDK6* and *BRAF* copy numbers were lower than the non-focal *MET* amplification group (*CDK6*: 2.11 copies vs. 2.48 copies, respectively, *p* = 0.0278; *BRAF*: 2.0 copies vs. 2.47 copies, respectively, *p* = 0.0086). *EGFR* copy number was not different between the focal *MET* amp and non-focal *MET* amp groups (2.27 copies vs. 2.44 copies, respectively; *p* = 0.421). This illustrated that a higher copy number in *MET* and low copy numbers in *CDK6* and *BRAF*, but not *EGFR*, were associated with focal *MET* amplification. 

Next, we investigated the proportions of focal vs. non-focal amplifications among different cancer types (Table 3). In the NSCLC cohort, 10 out of 144 patients (6.9%) had *MET* amplification. Among 10 patients with *MET* amplification, four had focal amplification, and six had non-focal amplification. The proportion of focal amplification in total *MET* amplification in the NSCLC cohort was 40%. The proportions of *MET* focal amplifications in gastrointestinal, breast, and other cancers were 25%, 50%, and 0%, respectively. Focal amplification was found in 7 out of 291 samples (2.4%), and the proportion of focal amplification among *MET* amp was 7 out of 24 samples (29.2%). However, the number of patients who had *MET* focal amplification was too small to elucidate any meaningful statistical conclusion. Further investigation is warranted in a larger patient cohort. 

Next, we examined whether there were differences in the coexistence of *EGFR* driver mutations between focal and non-focal *MET* amps in NSCLC patients. Among 17 NSCLC patients with *MET* alts, 10 had *MET* amp. Among them, four had focal *MET* amp, and six had non-focal. Co-occurrence of *EGFR* driver mutations was found in five *MET* amp patients, two with a focal amp. *EGFR* T790M was also present in both patients with focal *MET* amp and one patient with a non-focal amp. This is suggestive of acquired resistance to third-generation EGFR TKI [10], although the treatment histories of these patients are unknown.

Lastly, we compared our focal amplification definition to the amplification determined by a CLIA (Clinical Laboratory Improvement Amendments) certified laboratory at Guardant Health, Inc using an independent cohort. (original data; Table S1) In 734 patients tested after Sep/2018, our definition of focal amplification was detected in 31 out of 734 patients (4.2%). Guardant360 reported *MET* amplification in 30 patients (4.1%). The positive percent agreement (PPA; focal in both TMDU and G360/focal in G360), negative percent agreement (NPA; non-focal in both TMDU and G360/non-focal in G360), positive predictive value (PPV; focal in both TMDU and G360/focal in TMDU), and negative predictive value (NPV; non-focal in both TMDU and G360/non-focal in TMDU) were 83.3% (25/30), 98.7% (471/477), 80.6% (25/31), and 98.9% (471/476), respectively. Using our algorithm in the complete study cohort (test and validation sets combined, *n* = 1025), focal amplification of *MET* was found in 38 patients (3.7%).

## 4. Discussion

We describe a method for determining focal *MET* amplification using unfiltered data from comprehensive genomic profiling of cfDNA in advanced cancer patients. Focal *MET* amp was found in 3.7% (38/1025) of patients with diverse cancers and accounted for only ~30% of all *MET* amps. This distinction has clinical importance. Focal *MET* amplification is likely a driver alteration and, therefore, a therapeutic target. Non-focal amplification or aneuploidy is unlikely to be a driver alteration and, therefore, not an ideal therapeutic target.

In previous studies using tumor tissue, *MET* amp has been defined using the ratio of *MET* to CEP7 by FISH, and *MET* amps were found in up to ~5% of patients with NSCLC or gastric cancer [5]. In a phase I study, in which the definition of *MET* amp was as follows: “the *MET*: CEP7 signal ratio was ≥2.0 or when this ratio was <2.0, but there were >20 copies of *MET* signals in more than 10% of the tumor nuclei counted,” *MET* amp was detected in 2.5% of patients with advanced solid tumors in different cancer types. However, there was no significant difference in sensitivity to MET inhibitors between the *MET* amp group and the not amplified group [2]. Therefore, a higher focal *MET* amp was proposed as a threshold, and a correlation with the therapeutic effect was seen in patients whose *MET*/CEP was 5 or more [6,7,8]. This suggests that it is necessary to find a group with *MET*-only amplification, without aneuploidy of chromosome 7, to enhance the therapeutic effect of MET inhibitors. Our study showed that a higher *MET* copy number in plasma was more likely to be due to focal amplification. 

Furthermore, this result indicates that it is necessary to interrogate not only *MET* but also other adjacent genes on the same chromosome when designing companion diagnostic tests for *MET* amplification. Currently, a typical biomarker for amplification investigates only the target gene and does not include adjacent genes. However, this study illustrated that focal *MET* amplification was observed in only approximately 30% of “*MET* amplification,” and the rest were likely aneuploidy of chromosome 7. This distinction is critical for selecting a patient population in which *MET* amp drives cancer and may therefore be a target population for treatment with MET inhibitors. The idea of considering adjacent genes to identify focal gene amplification might be critical not only for *MET* but also for *FGFR1* and *FGFR2*, for which there are emerging targeted therapeutics. 

However, the algorithm needs to be optimized. We found several patterns of co-amplification of three genes on chromosome 7 (*BRAF*, *CDK6*, and *EGFR*) in this study and defined aneuploidy as having *CDK6* and/or *BRAF* amplified to the same degree. We defined the copy number of *CDK6* or *BRAF* “increasing together” as within ±0.5 of the *MET* copy number, but the condition of less than 0.5 may not have been necessary because even if the copy number of *CDK6* and *BRAF* is much higher (>0.5) than that of *MET*, it is still considered aneuploidy when all of them are high. For example, if our algorithm had excluded this rule, the NPA, PPV, and NPV would have been improved to 99.4% (from 98.7%), 89.3% (from 80.6%), and 99.0% (from 98.9%), respectively (PPA would have been same, 83.3%). Another point is whether or not to include *EGFR* in the definition. Unlike *MET*, *CDK6*, and *BRAF* genes on 7q, *EGFR* on 7p often behave independently, with apparently different values of copy number. Therefore, we removed *EGFR* from the definition of aneuploidy in this study. Further research is needed to validate this definition and its correlation with the clinical utility of MET targeting agents. Some of the MET tyrosine kinase inhibitors have been suggested or expected to have effects on *MET* amplification. For example, tepotinib and capmatinib, which are both approved by the FDA for NSCLC with *MET* exon14 skipping, have shown positive results for *MET* amplification. Improved anti activity of tepotinib plus gefitinib in patients with *EGFR*-mutant NSCLC and *MET* amplification was suggested [11], and limited efficacy of capmatinib in previously treated patients in advanced NSCLC with *MET* amplification was observed [12]. Savolitinib, a potent, selective MET TKI, plus osimertinib are undergoing trials to determine their effect on *EGFR* mutation-positive lung cancers with *MET* amplification [13]. Additionally, amivantamab is an EGFR–MET bispecific antibody with immune cell-directing activity that targets activating and resistant *EGFR* mutations and *MET* mutations and amplifications [14].

Recently, Lai and colleagues described *MET* amplification and polysomy (aneuploidy) using FISH and *MET*/CEP7 ratio in *EGFR* mutated NSCLC patients [15]. Among *MET* high group (defined by *MET* copy number equal to or more than 5.0 by FISH), *MET* amplification (defined by *MET*/CEP7 ratio equal to or more than 2.0) was observed only in 11.5%, and associated with suboptimal response to EGFR TKI, suggesting that *MET* amplification was another driver alteration, but it exists in limited population in *MET* high group. Roper and colleagues studied special and temporal heterogeneity of *MET* amplification in EGFR-positive NSCLC patients who received osimertinib [16]. In post-osimertinib biopsy samples, heterogeneity of *MET* amplification and polysomy were observed. Therefore, it is important to carefully interpret *MET* copy number gain result from the tissue-based assay. It may be ideal to use liquid biopsy to capture heterogeneity in the clinical setting, rather than multiple biopsied used in this study. Our study showed the feasibility of capturing *MET* focal amplification using liquid biopsy.

A major limitation of this study is the small sample size and the lack of complete clinical history, including treatment and response data. For example, in the *MET* alt dataset, we found NSCLC cases with *EGFR* driver and T790M with focal *MET* amp, suggesting resistance to EGFR TKI. It has been reported that NSCLC with secondary resistance to EGFR inhibitors tends to show *MET* amp [17,18]. However, we did not know the treatment history of the patients from whom the plasma was collected. Additionally, it is ideal for validating the proposed *MET* focal and non-focal amplification using FISH assays, the current golden standard to measure *MET* amplification. However, our de-identified data did not come with FISH results. Further study is warranted to validate the current proposed *MET* focal amplification definition by FISH assays in future studies.

## 5. Conclusions

This study describes an approach to distinguish focal and non-focal *MET* amplification using unfiltered data from comprehensive genomic profiling of cfDNA in advanced cancer patients. Focal *MET* amp accounted for ~30% of all *MET* amps, was found in 3.7% of patients with diverse cancers, and was associated with a higher plasma copy number. Clinical studies are warranted to assess the clinical utility of targeted therapies for tumors with focal *MET* amplification detected by NGS of cfDNA.

## Figures and Tables

**Table 1:** Demographic comparison of 1025 patients with or without *MET* alt.

Characteristics	Total Patients, *n* = 1025 (100%)	*MET* Alt Not Detected, *n* = 915 (89.3%)	*MET* Alt Detected, *n* = 110 (10.7%)	*p*-Values **
Mean age at time of testing, y	62.4	62.4	62.4	0.966
(CI 95%), *n* = 880 known	(61.6–63.3)	(61.5–63.3)	(59.4–65.3)	
Gender				0.00234
Men	482 (47.0%)	415 (86.1%)	67 (13.9%)	
Women	543 (53.0%)	500 (92.1%)	43 (7.9%)	
Type of cancer				0.0863
NSCLC	510 (49.8%)	448 (87.8%)	62 (12.2%)	
SCLC	9 (0.9%)	6 (66.7%)	3 (33.3%)	
Colorectal	77 (7.5%)	71 (92.2%)	6 (7.8%)	
Gastroesophageal	36 (3.5%)	29 (80.6%)	7 (19.4%)	
Other gastrointestinal	158 (15.4%)	147 (94.2%)	9 (5.8%)	
Breast	92 (9.0%)	84 (91.3%)	8 (8.7%)	
Unknown primary	20 (2.0%)	18 (94.7%)	1 (5.3%)	
Gynecologic	46 (4.5%)	43 (93.5%)	3 (6.5%)	
Prostate	32 (3.1%)	27 (84.4%)	5 (15.6%)	
Other	45 (4.4%)	42 (87.5%)	6 (12.5%) *	

* Includes patients with neuroblastoma (*n* = 2), neuroendocrine tumor (*n* = 2), melanoma (*n* = 1), and other (*n* = 1). ** *p*-values were calculated using the *t*-test for linear variables (age at time of testing), Fisher’s exact test (gender), and the chi-square test (type of cancer) for categorical variables.

**Table 2:** Patterns of copy number of four genes on chromosome 7.

Case	*EGFR*	*CDK6*	*MET*	*BRAF*	Feature	Focal/Non-Focal
1	2.2 *	2.2	2.1	2.3	*MET* copy number is lower than 2.2	non-focal
2	2.2	2.1	2.1	2.3		non-focal
3	2.2	2.2	2.1	2.0		non-focal
4	3.1	2.9	2.8	3	*MET* copy number is increased together with either *CDK6* and/or *BRAF*	non-focal
5	2.9	2.8	2.6	2.8		non-focal
6	2.6	2.6	2.6	2.5		non-focal
7	2.2	2.3	2.2	2.2		non-focal
8	2.8	3.6	3.3	3.6		non-focal
9	2.4	2.3	2.6	2.5		non-focal
10	2.0	2.6	2.8	2.6		non-focal
11	3	2.3	2.2	2.2		non-focal
12	3.3	2.3	2.3	2.3		non-focal
13	80	2.6	2.5	2.6		non-focal
14	2.0	2.5	2.3	2.4		non-focal
15	2.0	2.6	2.5	2.5		non-focal
16	2.1	2.0	2.3	2.2		non-focal
17	2.0	2.2	2.2	2.0		non-focal
18	3.2	2.0	3.3	2.0	*MET* gene is amplified without co-amplification of *CDK6* and *BRAF*	focal
19	2.5	2.0	2.5	2.0		focal
20	2.0	2.0	2.6	2.0		focal
21	2.0	2.5	3.7	2.0		focal
22	2.0	2.0	2.2	2.0		focal
23	2.2	2.3	6.2	N/A **		focal
24	2.0	2.0	2.3	2.0		focal

* Copy numbers 2.2 and above are shown in red. ** N/A represents data not available. Algorithm to determine focal amp: (a) *MET* copy number ≥ 2.2. (b) *MET* gene is amplified without co-amplification of *CDK6* and *BRAF*. Co-amplification status was defined as “increased together” when the copy number of other genes (*CDK6* or *BRAF*) ≥ 2.2, and the difference with *MET* amplification is within +/−0.5. (c) *MET* amplification that satisfies both (a) and (b) is defined as focal. Cases 18 and 19 are considered focal because co-amplification of *MET* occurred only with the *EGFR* gene but not with *CDK6* and *BRAF* genes. Cases 21 and 23 were defined as focal amplification because the difference in amplification magnitude between *MET* and *CDK6* was more than 0.5.

**Table 3:** Focal vs. non-focal *MET* amplification by cancer type in training set (*n* = 291).

Cancer Type (*n* Patients, %w/AMP)	Patients w/Focal *MET* Amp in ≥1 Sample	Patients w/Only Non-Focal *MET* Amp	Proportion Focal	*p*-Value
NSCLC (144, 6.9%)	4 (2.8%)	6 (4.2%)	4/10 (40.0%)	0.140
Gastrointestinal (69, 5.8%)	1 (1.4%)	3 (4.3%)	1/4 (25.0%)	
Breast (26, 15.4%)	2 (7.7%)	2 (7.7%)	2/4 (50.0%)	
Other (52, 11.5%)	0 (0.0%)	6 (11.5%)	0/6 (0%)	
Overall (291, 8.2%)	7 (2.4%)	17 (5.8%)	7/24 (29.2%)	

## Data Availability

Restrictions apply to the availability of these data. Data were obtained from Guardant Health Japan, Corp. and are available from the authors only with the permission of Guardant Health Japan, Corp.

## Supplementary Materials

The following are available online at https://www.mdpi.com/article/10.3390/curroncol28050317/s1, Figure S1: Representative examples of copy number plots showing focal (top pane) vs. non-focal (bottom pane) *MET* amplification. Table S1: Original CNs of 3 genes after September 2018.

## References

[1] Organ S.L., Tsao M.-S. (2011). An overview of the c-MET signaling pathway. Ther. Adv. Med. Oncol..

[2] Jardim D.L., Tang C., Gagliato D.D., Falchook G.S., Hess K., Janku F., Fu S., Wheler J.J., Zinner R.G., Naing A. (2014). Analysis of 1,115 Patients Tested for MET Amplification and Therapy Response in the MD Anderson Phase I Clinic. Clin. Cancer Res..

[3] Schrock A.B., Frampton G.M., Suh J., Chalmers Z.R., Rosenzweig M., Erlich R.L., Halmos B., Goldman J., Forde P., Leuenberger K. (2016). Characterization of 298 Patients with Lung Cancer Harboring MET Exon 14 Skipping Alterations. J. Thorac. Oncol..

[4] Frampton G.M., Ali S.M., Rosenzweig M., Chmielecki J., Lu X., Bauer T.M., Akimov M., Bufill J.A., Lee C., Jentz D. (2015). Activation of MET via Diverse Exon 14 Splicing Alterations Occurs in Multiple Tumor Types and Confers Clinical Sensitivity to MET Inhibitors. Cancer Discov..

[5] Kawakami H., Okamoto I., Okamoto W., Tanizaki J., Nakagawa K., Nishio K. (2014). Targeting MET Amplification as a New Oncogenic Driver. Cancers.

[6] Caparica R., Yen C.T., Coudry R., Ou S.-H.I., Varella-Garcia M., Camidge D.R., de Castro G. (2017). Responses to Crizotinib Can Occur in High-Level MET -Amplified Non–Small Cell Lung Cancer Independent of MET Exon 14 Alterations. J. Thorac. Oncol..

[7] Tong J.H., Yeung S.F., Chan A.W.H., Chung L.Y., Chau S.L., Lung R.W.M., Tong C.Y., Chow C., Tin E.K.Y., Yu Y.H. (2016). MET Amplification and Exon 14 Splice Site Mutation Define Unique Molecular Subgroups of Non–Small Cell Lung Carcinoma with Poor Prognosis. Clin. Cancer Res..

[8] Noonan S.A., Berry L., Lu X., Gao D., Barón A.E., Chesnut P., Sheren J., Aisner D.L., Merrick D., Doebele R.C. (2016). Identifying the Appropriate FISH Criteria for Defining MET Copy Number–Driven Lung Adenocarcinoma through Oncogene Overlap Analysis. J. Thorac. Oncol..

[9] Ikeda S., Schwaederle M., Mohindra M., Jardim D.L., Kurzrock R. (2018). MET alterations detected in blood-derived circulating tumor DNA correlate with bone metastases and poor prognosis. J. Hematol. Oncol..

[10] Leonetti A., Sharma S., Minari R., Perego P., Giovannetti E., Tiseo M. (2019). Resistance mechanisms to osimertinib in EGFR-mutated non-small cell lung cancer. Br. J. Cancer.

[11] Wu Y.-L., Cheng Y., Zhou J., Lu S., Zhang Y., Zhao J., Kim D.-W., Soo R.A., Kim S.-W., Pan H. (2020). Tepotinib plus gefitinib in patients with EGFR-mutant non-small-cell lung cancer with MET overexpression or MET amplification and acquired resistance to previous EGFR inhibitor (INSIGHT study): An open-label, phase 1b/2, multicentre, randomised trial. Lancet Respir. Med..

[12] Wolf J., Seto T., Han J.Y., Reguart N., Garon E.B., Groen H.J., Tan D.S., Hida T., de Jonge M., Orlov S.V. (2020). Capmatinib in MET Exon 14-Mutated or MET-Amplified Non-Small-Cell Lung Cancer. N. Engl. J. Med..

[13] Sequist L.V., Han J.-Y., Ahn M.-J., Cho B.C., Yu H., Kim S.-W., Yang J.C.-H., Lee J.S., Su W.-C., Kowalski D. (2020). Osimertinib plus savolitinib in patients with EGFR mutation-positive, MET-amplified, non-small-cell lung cancer after progression on EGFR tyrosine kinase inhibitors: Interim results from a multicentre, open-label, phase 1b study. Lancet Oncol..

[14] Yun J., Lee S.-H., Kim S.-Y., Jeong S.-Y., Kim J.-H., Pyo K.-H., Park C.-W., Heo S.G., Yun M.R., Lim S. (2020). Antitumor Activity of Amivantamab (JNJ-61186372), an EGFR-cMet Bispecific Antibody, in Diverse Models of EGFR Exon 20 Insertion-Driven NSCLC. Cancer Discov..

[15] Lai G.G.Y., Lim T.H., Lim J., Liew P.J.R., Kwang X.L., Nahar R., Aung Z.W., Takano A., Lee Y.Y., Lau D.P.X. (2019). Clonal MET Amplification as a Determinant of Tyrosine Kinase Inhibitor Resistance in Epidermal Growth Factor Receptor–Mutant Non–Small-Cell Lung Cancer. J. Clin. Oncol..

[16] Roper N., Brown A.-L., Wei J.S., Pack S., Trindade C., Kim C., Restifo O., Gao S., Sindiri S., Mehrabadi F. (2020). Clonal Evolution and Heterogeneity of Osimertinib Acquired Resistance Mechanisms in EGFR Mutant Lung Cancer. Cell Rep. Med..

[17] Bean J., Brennan C., Shih J.-Y., Riely G., Viale A., Wang L., Chitale D., Motoi N., Szoke J., Broderick S. (2007). MET amplification occurs with or without T790M mutations in EGFR mutant lung tumors with acquired resistance to gefitinib or erlotinib. Proc. Natl. Acad. Sci. USA.

[18] Engelman J.A., Zejnullahu K., Mitsudomi T., Song Y., Hyland C., Park J.O., Lindeman N., Gale C.-M., Zhao X., Christensen J. (2007). MET Amplification Leads to Gefitinib Resistance in Lung Cancer by Activating ERBB3 Signaling. Science.

