# Supplementary material for: Comprehensive Genomic Profiling of Circulating Cell-Free DNA Distinguishes Focal MET Amplification from Aneuploidy in Diverse Advanced Cancers
Source: Curr Oncol. 2021 Sep 26;28(5):3717–28. doi: 10.3390/curroncol28050317 (PMC8534719; doi:10.3390/curroncol28050317)
Supplement: Supplementary file 1 [file curroncol-28-00317-s001.zip › curroncol-1329116-supplementary.pdf]

Current Oncology

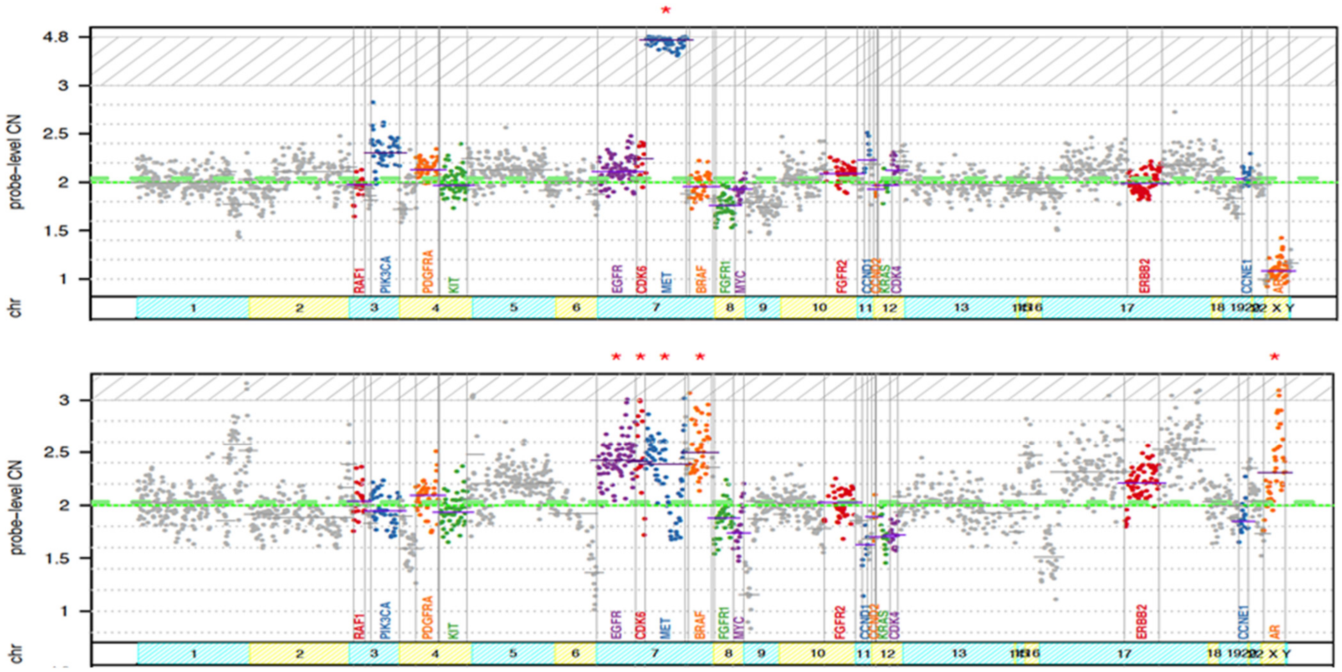

Figure S1. Representative examples of copy number plots showing focal (top pane) vs. non-focal (bottom pane) *MET* amplification In the top pane, only the *MET* gene is amplified, indicating focal. In the bottom pane, other genes on chromosome 7 are amplified simultaneously with *MET*, indicating non-focal amplification.
